# Mechanisms of Severe Adductor Longus Injuries in Professional Soccer Players: A Systematic Visual Video Analysis

**DOI:** 10.1177/23259671241309647

**Published:** 2025-02-06

**Authors:** Aleksi Jokela, Giulio Pasta, Francesco Della Villa, Arnaldo Abrantes, Dimitrios Kalogiannidis, Alvaro García-Romero-Pérez, Marco Marano, Dmitriy Skibinskyi, Stefano Mazzoni, Ricard Pruna, Xavier Valle, Lasse Lempainen

**Affiliations:** * Department of Orthopaedics and Traumatology, Turku University Hospital, Turku, Finland; † Department of Physical Activity and Health, Paavo Nurmi Centre, University of Turku, Turku, Finland; ‡ Parma Calcio, Parma, Italy; § Education & Research Department, Isokinetic Medical Group, FIFA Medical Centre of Excellence, Bologna, Italy; ‖ Aston Villa FC, Birmingham, United Kingdom; ¶ Chelsea FC, London, United Kingdom; # Watford FC, Watford, United Kingdom; ** Clinica Ars Medica, FC Lugano, Lugano, Switzerland; †† FC Shakhtar Donetsk, Donetsk, Ukraine; ‡‡ AC Milan, Milan, Italy; §§ FC Barcelona, Medical Services, FIFA Centre of Excellence, Barcelona, Spain; |||| ICATME, Hospital Universitari Dexeus, UAB, Barcelona, Spain; ¶¶ FinnOrthopaedics/Hospital Pihlajalinna, Turku, Finland; ## Ripoll y De Prado, FIFA Medical Centre of Excellence, Madrid, Spain; a Department of Physical Activity and Health, Paavo Nurmi Centre, University of Turku, Turku, Finland; b Aspetar Orthopaedic and Sports Medicine Hospital, Doha, Qatar; Investigation performed at FinnOrthopaedics/Hospital Pihlajalinna, Turku, Finland

**Keywords:** adductor longus, injury mechanism, video analysis, muscle-tendon injuries

## Abstract

**Background:**

Changing direction, kicking, reaching, and jumping have been found to be the primary mechanisms of adductor longus injury. No previous studies specifically analyzing severe adductor longus injury mechanisms using video analysis have been published.

**Purpose:**

To systematically analyze video footage to describe the mechanisms of severe acute adductor longus injuries in male professional soccer players.

**Study Design:**

Cross-sectional study; Level of evidence, 3.

**Methods:**

A total of 20 professional male soccer players (median age, 27 years; range, 18-35 years) who experienced an acute adductor longus injury during a match between October 2017 and December 2023 were included. All analyzed injuries were severe, either complete adductor longus tendon ruptures or partial lesions resulting in an absence from soccer competition of >28 days. Two authors independently reviewed the injuries based on a comprehensive injury causation model. Factors analyzed included playing situation, player/opponent behavior, and biomechanical descriptions encompassing whole-body and joint movements/positions.

**Results:**

Of the 20 included injuries, 13 (65%) were considered noncontact and 7 (35%) were indirect contact. A closed kinetic chain (CKC) injury mechanism was found in 14 injuries (70%), an open kinetic chain (OKC) mechanism was found in 3 injuries (15%), and the injury occurred during high-speed running in the remaining 3 cases (15%). Player actions at the time of injury included reaching with the uninjured leg (CKC stretching; n = 11 [55%]), reaching with the injured leg (OKC stretching; n = 2 [10%]), dribbling (n = 2 [10%]), and landing (n = 2 [10%]). In CKC injuries, hip extension, hip abduction, and external rotation were all found in 64% of the cases. All OKC injuries involved hip abduction, external rotation, and rapid change of movement from hip extension to flexion.

**Conclusion:**

Severe adductor longus injuries occurred predominantly during CKC actions, particularly when reaching for the ball with the uninjured leg. These injuries were consistently characterized by a combination of hip extension, abduction, and external rotation. A crucial aspect in these injuries appears to be the involvement of an eccentric muscle action, featuring rapid muscle activation during rapid muscle lengthening.

Groin injuries are prevalent among soccer players, often resulting in prolonged absences because of their severity.^17^ Hip adductor injuries constitute 23% of all muscle injuries; thus, a squad of 25 players can expect to experience 2 to 4 acute adductor injuries per season.^7,25^ The most frequently affected muscle in acute groin injuries is the adductor longus, which originates at the pubic body and converges with the insertion of the rectus abdominis.^1,22,23^

Several risk factors have been recognized in acute adductor longus injuries, including previous acute groin injuries, adductor weakness, any injury sustained in the preceding season, and reduced rotational hip range of motion.^8^ While most adductor longus injuries respond well to nonoperative treatment, some severe cases may require surgical intervention, particularly among professional soccer players.^8,14^ Previous studies have considered muscle injuries severe if they caused >28 days of absence from soccer play.^5,7^ Complete adductor tears cause a mean absence of 8.9 weeks in patients treated nonoperatively and 14.2 weeks in patients treated surgically.^8^ A complete adductor longus tendon rupture with significant retraction is often an indication for surgery.^8^ However, conflicting results exist regarding whether the tendon-bone gap is a negative prognostic factor.^8^

Enhancing our understanding of injury mechanisms and related factors is crucial to developing prevention, treatment, and return-to-play strategies. To date, only 1 study, conducted by Serner et al,^21^ has specifically investigated adductor longus injury mechanisms using video analysis. The authors examined injuries in 17 professional soccer players and revealed that the injuries occurred during either a closed kinetic chain (CKC) movement, where the injured leg moved without touching the ground, or an open kinetic chain (OKC) movement, where the injured leg had contact with the ground as the pelvis/trunk moved, implicating that typical biomechanical factors were involved in injury. The research group included all adductor longus injuries, regardless of severity.^21^

Recent studies utilizing video analysis, such as the work by Della Villa et al,^5^ have documented increased research on injury mechanisms, adding significant value to understanding muscle-tendon injuries in soccer. Their analysis of 103 severe muscle injuries in the lower limbs indicated that 63% of adductor injuries resulted from noncontact situations.

Severe muscle-tendon injuries lead to substantial time loss from sports and can seriously affect team performance and players’ careers. Therefore, a comprehensive understanding of the mechanisms behind these specific injuries is pivotal for successful management and prevention strategies. The purpose of this study was to systematically analyze video footage to describe the mechanisms of severe acute adductor longus injuries in male professional soccer players.

## Methods

According to the Medical Research Act (No. 488/1999), no ethical review was necessary or required beforehand, as the study did not use invasive methods. All included participants were informed about the study setup, they participated on a voluntary basis, and consent was acquired from all athletes at inclusion according to the Declaration of Helsinki. All video images used as examples were anonymized (ie, the faces and identifying information were blurred).

Professional male soccer players who experienced an acute adductor longus injury were recruited from 2 specialized sports medicine departments between October 2017 and December 2023. Inclusion criteria encompassed professional male soccer players aged 18 to 40 years who encountered sudden groin pain during a match. Additionally, the players had an adductor longus injury confirmed on magnetic resonance imaging (MRI) conducted within 7 days of the injury date, along with accessible video footage capturing the moment of injury. Experienced musculoskeletal radiologists (including G.P.) analyzed the MRI scans, and the diagnoses were confirmed by the corresponding author (L.L.). T1-, T2-, and proton density–weighted coronal, sagittal, and axial views were routinely performed in all patients. The strength of the MRI device was 1.5 to 3.0 T. Injured players were from different countries, so all MRI scans were not performed on the same scanner. All injuries included in the study were severe, either complete adductor longus tendon ruptures or partial lesions resulting in an absence from soccer competition of >28 days.^5,7^ Exclusion criteria involved injuries that occurred from nonmusculotendinous causes, inadequate video quality, or refusal to provide video footage.

### Video Acquisition and Processing

We obtained videos either from public sources or through training crew–managed archives. Videos were stored in MP4 format with standard quality. Using iMovie Version 10.1.12 software, we edited injury sequences and converted them into QuickTime (.mov) format, allowing frame-by-frame navigation via QuickTime Player Version 10.4. The video processing procedures followed the steps presented in a prior study by Serner et al.^21^ The footage was edited to display the period from the beginning of the performance before the injury to its cessation after injury. Furthermore, shorter clips of the injury moment from different camera angles were created, resulting in 1 full situation clip and 1 to 4 additional slow-motion clips.

### Determination of Injury Movement

The determination of injury movement and moment was based on interviews with the athletes (within 48 hours in the majority of the cases), injury mechanism, body positions, and athlete reactions.

### Video Analysis

Two authors from the field of sports medicine (L.L. and A.J.), an orthopaedic surgeon and a medical doctor experienced in video analysis, independently evaluated all videos in real time, slow motion, and frame by frame to describe specific adductor longus injury mechanisms. The analysts used their personal computers with a video player enabling repeated viewings. Similar to the methodology in the study by Serner et al,^21^ the analysts independently reviewed injuries, answering open-ended questions detailing patterns during the injury moment, based on a comprehensive injury causation model.^2^ Factors analyzed included playing situation, player/opponent behavior, and biomechanical descriptions encompassing whole-body and joint movements/positions.

A standardized scoring form was used.^9^ Additionally, the injury-inciting movements were categorized into either an OKC movement, a CKC movement, or “unsure” if the previously described categorization was inconclusive. The level of contact during the injury situation was divided into direct contact (contact to the injured area), indirect contact (contact to other body part, such as trunk, shoulder, or uninjured leg), and noncontact (no contact, with the closest opponent ±2 m away at the time of injury). Both analysts scored the videos independently, unaware of each other’s assessments. Any discrepancies were resolved in a consensus meeting during which videos were reviewed again until agreement was achieved. If agreement could not be achieved in the consensus meeting, a third reviewer (X.V.) was invited to settle disagreements. Visual estimations of joint positions were included in the analysis. However, scoring some variables (pelvis and ankle) was challenging, leading to their exclusion from the assessment. Additionally, only knee joint angles were analyzed, with an accuracy of 45°. We also considered the minute zone of match play when the injury occurred (0-15, 15-30, 30-45+, 45-60, 60-75, or 75-90+ minutes). Scoring was conducted using Excel 2018 (Version 16.16.27, Microsoft Corp).

## Results

### Participants

A total of 20 male professional soccer players with severe acute adductor longus injury were included (median age, 27 years; range, 18-35 years). The study population involved 1 goalkeeper, 8 defenders, 7 forwards, and 4 midfielders. There were 16 complete and 4 partial proximal adductor longus tendon ruptures.

### Injury Mechanisms and Patterns

**Figure 1.**
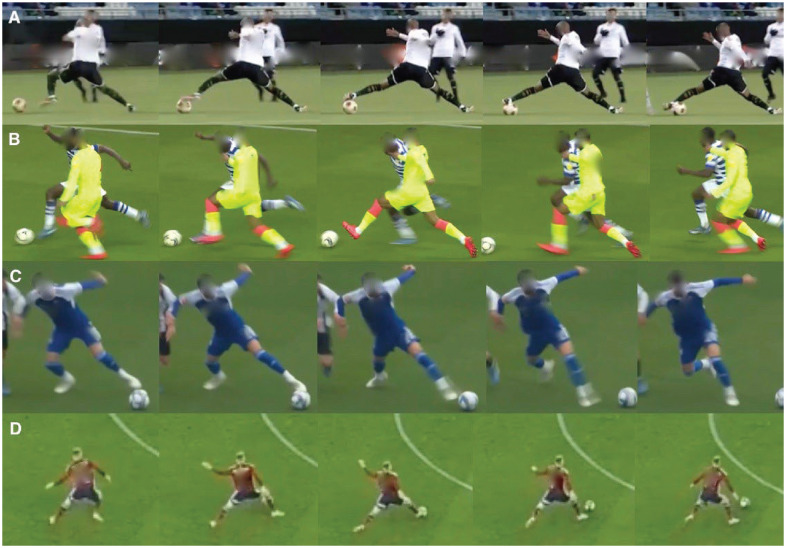
(A-D) Four examples of the predominant pattern: reaching with the uninjured leg (closed kinetic chain stretching).

**Figure 2.**
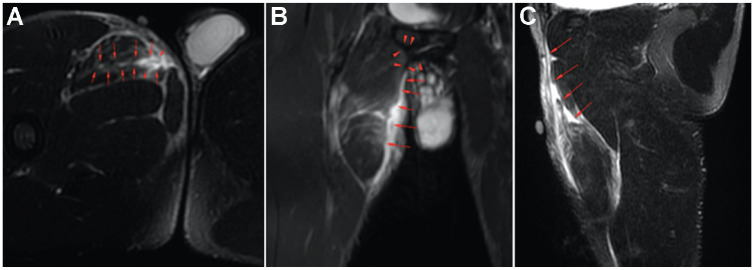
Examples of magnetic resonance imaging findings on (A) axial T2-weighted, (B) coronal short-tau inversion recovery (STIR), and (C) sagittal T2-weighted views after a closed kinetic chain stretching injury: acute proximal myotendinous adductor longus tear, with large connective tissue affected, aponeurotic gap and wavy remaining aponeurosis, secondary to complete rupture and retraction of the intramuscular aponeurosis. The red arrows show the lesion area. In the lesion area, there is an irregular complete transverse oblique rupture of the intramuscular aponeurosis, with aponeurotic retraction. The aponeurotic rupture involves the proximal third of the intramuscular aponeurosis, located 25 mm distal to the right pubic bone. The aponeurotic gap is 6 cm in longitudinal diameter and 4 cm in transverse diameter. The adductor longus muscle belly shows loss of tension and architectural distortion. Imaging technique: sagittal and axial T2-weighted fat-saturated (FS) sequences; axial, coronal, and sagittal T1-weighted and STIR sequences; axial proton-density (PD) sequences; and PDFS sequences.

Descriptive information on the injuries is presented in Table 1. There were 13 injuries (65%) classified as noncontact, while 7 (35%) were classified as indirect contact. The CKC injury mechanism was found in 14 cases (70%), while OKC injuries were found in 3 (15%). The remaining 3 injuries (15%) occurred during high-speed running, making categorization into open or closed kinetic action impossible. Player actions at the time of injury were categorized as reaching with the uninjured leg (CKC stretching) in 11 injuries (55%), reaching with the injured leg (OKC stretching) in 2 injuries (10%), dribbling in 2 injuries (10%), and landing in 2 injuries (10%). Additionally, the following actions were present (n = 1 for each): pressing (injured player applying pressure on the player who was in possession), jumping, and running (acceleration). Four examples of the predominant pattern, reaching with the uninjured leg (CKC stretching), are presented in Figure 1. An example of MRI findings is presented in Figure 2.

**Table 1:** Descriptive Information on Injuries (N = 20)^*a*^

Variable	Value
Team action	
Defensive	10 (50)
Offensive	10 (50)
Player contact	
Direct	0 (0)
Indirect (upper limb/trunk/uninjured leg)	7 (35)
None (opponent <2 m away)	12 (60)
None (opponent >2 m away)	1 (5)
Foul play	
No foul	19 (95)
Foul	1 (5)
Balance	
Player out of balance	5 (25)
Movement speed	
Maximal sprinting	6 (30)
Running	14 (70)
Minute zone of match play	
0-15	3
15-30	5
30-45+	3
45-60	2
60-75	6
75-90+	1

a Data are presented as No. of injuries, with values in parentheses representing percentages.

### Biomechanical Findings

In CKC injuries, hip extension, hip abduction, and external rotation were all found on the injured side at the time of injury in 64% of the cases. All OKC injuries involved hip abduction, external rotation, and rapid change of movement from hip extension to flexion (Table 2). Biomechanical body positions at the time of injury are detailed in Table 3. The most common findings on the injured-side hip were sagittal extension, frontal abduction, and external rotation. The injured-side knee was typically at <45° of flexion. Trunk position involved a lot of variability.

**Table 2:** Injury Categorization and Movement Description^*a*^

Injury No.	Player Action at Injury	Rapid Movement Change From Hip Extension to Flexion	Rapid Movement Change From Hip Abduction to Adduction	Hip Externally Rotated	Ball Impact
OKC					
1	Reaching (with the injured leg)	Yes	Yes	Yes	Yes
2	Landing	Yes	Yes	Yes	No
3	Reaching (with the injured leg)	Yes	No	Yes	Yes
CKC		Hip extension	Hip abduction	Hip externally rotated	
4	Reaching (with the uninjured leg)	Yes	Yes	Yes	—
5	Reaching (with the uninjured leg)	Yes	Yes	Yes	—
6	Reaching (with the uninjured leg)	No	Yes	Yes	—
7	Pressing	Yes	Yes	Yes	—
8	Reaching (with the uninjured leg)	Yes	Yes	Yes	—
9	Reaching (with the uninjured leg)	Yes	Yes	Yes	—
10	Jumping	No	Yes	Yes	—
11	Landing	Yes	Yes	Yes	—
12	Reaching (with the uninjured leg)	Yes	Yes	Yes	—
13	Reaching (with the uninjured leg)	Yes	Yes	No	—
14	Reaching (with the uninjured leg)	Yes	No	No	—
15	Reaching (with the uninjured leg)	Yes	Yes	Yes	—
16	Reaching (with the uninjured leg)	Yes	No	Yes	—
17	Reaching (with the uninjured leg)	Yes	Yes	Yes	—
Unsure					
18	Dribbling	—	—	—	—
19	Running (acceleration)	—	—	—	—
20	Dribbling	—	—	—	—

a Dashes indicate areas not applicable. CKC, closed kinetic chain; OKC, open kinetic chain.

**Table 3:** Biomechanical Descriptions of the Body Position at the Time of Injury^*a*^

Player No.	Trunk Flexion or Rotation, Sagittal/Frontal/Transverse^*b*^	Hip Rotation, Sagittal/Frontal/Transverse		Knee, Sagittal	
		Injured Side	Uninjured Side	Injured Side	Uninjured Side
OKC					
1	Neutral/neutral/neutral	Flex/abd/ER	Ext/abd/ER	Flex <45°	Flex <45°
2	Flex/toward/toward	Flex/abd/ER	Flex/abd/ER	Flex <45°	Flex <45°
3	Flex/away/neutral	Flex/abd/ER	Ext/abd/ER	Flex <45°	Flex <45°
CKC					
4	Ext/toward/toward	Ext/abd/ER	Flex/neutral/neutral	Flex <45°	Flex 45°-90°
5	Neutral/neutral/away	Ext/abd/ER	Flex/abd/neutral	Flex <45°	Flex <45°
6	Flex/neutral/away	Flex/abd/ER	Flex/abd/neutral	Flex 45°-90°	Flex <45°
7	Ext/away/away	Ext/abd/ER	Flex/abd/ER	Flex <45°	Flex 45°-90°
8	Flex/toward/toward	Ext/abd/ER	Flex/neutral/neutral	Flex <45°	Flex 45°-90°
9	Flex/neutral/neutral	Ext/abd/ER	Flex/abd/ER	Flex <45°	Flex 45°-90°
10	Neutral/neutral/neutral	Flex/abd/ER	Flex/abd/ER	Flex <45°	Flex 45°-90°
11	Flex/toward/away	Ext/abd/ER	Flex/abd/ER	Flex <45°	Flex 45°-90°
12	Flex/toward/neutral	Ext/abd/ER	Flex/abd/ER	Flex <45°	Flex 45°-90°
13	Neutral/neutral/neutral	Ext/abd/neutral	Flex/abd/ER	Flex <45°	Flex <45°
14	Ext/toward/away	Ext/neutral/neutral	Flex/abd/neutral	Flex <45°	Flex <45°
15	Flex/toward/away	Ext/abd/ER	Flex/abd/IR	Flex <45°	Flex <45°
16	Neutral/neutral & neutral	Ext/neutral/ER	Flex/abd/neutral	Flex <45°	Flex <45°
17	Flex/toward/away	Ext/abd/ER	Flex/abd/neutral	Flex <45°	Flex <45°
Most frequent positions	Flex/toward/away & neutral 53%/47%/41% & 41%	Ext/abd/ER 71%/88%/88%	Flex/abd/ER 88%/88%/53%	Flex <45° 94%	Flex <45° 59%

a Abd, abduction; CKC, closed kinetic chain; ER, external rotation; Ext, extension; Flex, flexion; IR, internal rotation; OKC, open kinetic chain.

b Related to injured side.

## Discussion

In this systematic visual video analysis study of severe and acute adductor longus injuries in soccer, we observed that most injuries occurred during CKC actions. The injured side commonly involved hip extension, abduction, external rotation, and knee extension.

### Injury Mechanisms

The mechanisms of injury observed shared similarities with previous studies investigating adductor injuries.^5,21^ While reaching actions were also identified in other studies, changes of direction and kicking were more prevalent injury mechanisms. In our study, reaching for the ball accounted for the highest occurrence at 65% of cases. Discrepancies between studies might be attributed to differences in injury severity. Serner et al^21^ encompassed all patients with acute onset of groin pain during soccer, while we specifically included only severe cases, complete adductor longus tendon ruptures, and partial lesions resulting in >28 days off from soccer. Most adductor injuries are mild,^7,17^ suggesting that only a few cases were severe in the study performed by Serner et al. Despite focusing on the same muscle, varying injury mechanisms between studies may be partially explained by severity differences.

From a biomechanical perspective, the injured-side hips typically displayed extension, abduction, and external rotation, while the injured-side knees were commonly at <45° of flexion. On the uninjured side, hips were often in flexion, abduction, and external rotation. There was more variability in trunk positions and uninjured-side knee flexion angles. These findings are consistent with previous results.^21^ In hip extension, abduction, and external rotation, the adductor muscles are in a lengthened position. Additionally, adductor injuries have been associated with rapid muscle lengthening and eccentric actions.^21^ Hence, these biomechanical factors likely play pivotal roles in adductor longus injury mechanisms.

No clear kicking injury mechanisms were observed in this study. However, previous studies have emphasized kicking as a significant factor in adductor longus injuries.^5,21^ Serner et al^21^ reported various kick actions causing adductor longus injuries, not only shots but also short and long passes. These movements typically involved diagonal actions with hip extension to flexion, hip abduction to adduction, and external hip rotation.^15,21^ Adductors are more engaged in pass kick actions than in in-step kicks, where the rectus femoris predominantly functions.^13,15^ Compared with previous studies, our study included more severe injuries, as there were 16 complete adductor longus ruptures, and 11 injuries underwent surgery. We did not report kicking injuries, so it can be speculated that kicking and passing may cause less severe injuries to the adductor longus. However, the sample size was relatively small, so wide generalizations can not be made.

In this study, we found that sprinting can cause severe adductor longus injuries. In 3 players, the injury moment could not be categorized into either OKC or CKC movement, as the injuries occurred during dribbling or acceleration. Overall, the movement speed was “maximal sprinting” in 30% and “running” in 70% of the cases. These findings suggest that high-speed running has a significant role in severe adductor longus injuries. Chaudhari et al^4^ measured adductor activation during run-to-cut maneuvers and compared the values between different compression shorts. They found that the ipsilateral adductor longus activates in all 5 stance phases, with peak activation occurring in the weight acceptance phase. During run-to-cut maneuvers, the hip adducts just before the contact, followed by an external hip abduction during the early and middle stances, during which the hip remains isometric. During late stance and early swing, the hip adducts again.^12^ All these actions and motions utilize the hip adductors, putting a lot of demand on the adductor longus. Therefore, rapid movements during high-speed sprints rationally can play a significant role in adductor longus injury mechanisms.

Eleven injuries occurred in the first half and 9 in the second half. The most common minute zones were 15 to 30 minutes (5 injuries) and 60 to 75 minute (6 injuries). Previous studies have reported a higher prevalence of adductor injuries in the first half of match play.^5,21^ However, Ekstrand et al^7^ reported a similar amount of injuries in both halves in their large cohort study, which included approximately 3000 muscle injuries.

The typical injury situations were rapid, often with the player focusing on the opponent giving pressure and/or on the ball. However, the key factor in these injuries appeared to be the rapid loading of the muscle-tendon unit during rapid lengthening. A significant number of injuries occurred during rapid reaching movements affecting the back leg. Although the movement was usually balanced and controlled, the adductor longus of the back leg had to tolerate rapid lengthening during quick ball-focused situations. The opponent was commonly within 2 m, adding pressure, which increased the number of distractive factors. We noted that poor touches or passes from teammates often preceded these movements. While these situations are inherent in soccer and cannot be entirely avoided, attention could potentially be directed toward enhancing body control, range of motion, and adductor strength and implementing injury-prone patterns in training programs to simulate real in-play situations.

### Prevention

In this study, rapid eccentric muscle contraction played a significant role in severe adductor longus injury mechanisms. Previous studies have suggested that strengthening the adductor longus to tolerate rapid loading in a lengthened state is crucial in preventing injuries.^21^ Therefore, eccentric training could be recommended as the cornerstone of preventing severe adductor longus injuries. Adductor strengthening has shown promising results in injury prevention. Two nonrandomized controlled trial intervention studies demonstrated a significant decrease in adductor injury incidence.^19,24^ Núñez et al^19^ reported an acute adductor injury rate of 0.07 per 1000 hours in the intervention group compared with 0.27 per 1000 hours in the control group. They also found adductor injuries occurring when between-leg adductor power asymmetry exceeded 10% and adductor-to-abductor power ratios were <0.9. Additionally, Tyler et al^24^ implemented an adductor strength program for at-risk athletes, resulting in a significant decrease in the incidence of adductor injuries (9% vs 38%). Two randomized controlled trials studied the effect of an adductor strengthening program on injury prevention in amateur soccer players^9,11^; however, they presented overall groin injury rates without specifying acute adductor injury rates. Hölmich et al^11^ found no significant difference between groups, whereas Harøy et al^9^ reported a higher mean weekly prevalence of groin injuries in the control group compared with the intervention group. Although more research is needed, adductor strengthening programs can be highly recommended for both performance training and injury prevention.

Understanding typical injury mechanisms and patterns should guide the development of prevention strategies, particularly in adductor injuries. Previous injury, adductor weakness, and reduced hip range of motion are associated with adductor longus injury risk,^8^ necessitating consideration in sport-specific training. It is strongly advised to incorporate prevention and rehabilitation strategies specific to the sport, tailored to the individual, and focused on addressing the underlying mechanisms of the injury.

The role of synergist muscles can be crucial in preventing adductor longus injury. The load on the adductor longus is significant in the situations described in this study. Alongside sport-specific training and eccentric strengthening of the adductor muscles, developing synergist muscles can reduce the load on the adductor longus. Considering the fact that injury mechanisms and situations are multifactorial and exhibit considerable variance, emphasis should be placed on large group synergist muscles, including hip flexors, knee extensors, trunk rotators, hip extensors, hip abductors, and trunk lateral flexors.^3,6,10,12,16,18,20^

### Limitations

Our study encountered several limitations. First, determining the exact moment of injury relied on athlete recall, injury mechanism assessment, and player reaction. It is important to note that there remains uncertainty regarding whether the injury occurred precisely at the defined moment. However, we mitigated this by discussing injuries with the players and confirming injuries through MRI scans, thus strengthening the reliability of the study outcomes.

Second, the sample size in our study was relatively small. This limited number of cases reduces the generalizability of our findings. Nonetheless, it is worth noting that our study constitutes the largest group in the literature analyzing adductor injuries through video analysis. For instance, Serner et al^21^ examined 17 injuries and Della Villa et al^5^ studied 16 adductor injuries. This highlights the critical necessity for more video analysis studies in the field, using systematic methods and larger sample sizes. Additionally, we included only severe injuries in our analysis. The injury mechanisms may be different for more minor injuries. Also, considering the study’s observational nature, other confounding variables or factors influencing injury mechanisms, such as player fitness levels, prior injury history, or specific game situations, were not fully accounted for and could potentially influence the outcomes.

Lastly, our reliance on visual video analysis presents an explicit limitation. This analysis was subject to the interpretations of the study authors and was influenced by video quality and the availability of camera views. Consequently, given the inherent limitations of visual video analysis, we categorized joint knee angles as <45°, 45° to 90°, and >90° (Table 3). Additionally, assessing other biomechanical body positions was conducted without angle estimations. Finally, while our study has shed light on these injury mechanisms, further research exploring the complicated biomechanical details and contextual factors surrounding these injury scenarios is needed.

## Conclusion

The mechanisms behind severe adductor longus injuries in soccer exhibited significant variability. Predominantly, these injuries occurred during CKC actions, particularly when reaching for the ball with the uninjured leg. The injuries were consistently characterized by a combination of hip extension, abduction, and external rotation, often accompanied by the ipsilateral knee positioned in extension or with slight flexion. A crucial aspect in understanding these severe and acute adductor longus injury mechanisms appears to be the involvement of an eccentric muscle action, featuring rapid muscle activation during rapid muscle lengthening.
